# Bilateral Deficit Using a Climbing-Specific Fingerboard Test and Its Association with Sport Climbers’ Performance

**DOI:** 10.3390/sports14070276

**Published:** 2026-07-03

**Authors:** Fernando Vilela, Amilton Vieira, Rafael Kons, Ubiratan Contreira Padilha, Denis César Leite Vieira, Martim Bottaro

**Affiliations:** 1 Strength and Conditioning Research Laboratory, College of Physical Education, University of Brasília, Brasília 70910-900, Brazil; fernasvf@gmail.com (F.V.); acmribeirao@gmail.com (A.V.); ucpadilha@outlook.com (U.C.P.); denisclvieira@hotmail.com (D.C.L.V.); 2 Department of Physical Education, Federal University of Bahia, Salvador 40170-110, Brazil; rafael.kons@ufba.br; 3 Human Physiology and Sports Physiotherapy Research Group (MFYS), Vrije Universiteit Brussel, 1050 Brussels, Belgium; 4 Cognition Action and Sensorimotor Plasticity (INSERM U1093 CAPS), Universite Bourgogne Europe, UFR des Sciences du Sport, Centre d’Expertise de la Performance, 21000 Dijon, France

**Keywords:** muscle strength, endurance capacity, fatigue resistance index, maximal isometric voluntary contraction, handgrip strength

## Abstract

This study aimed to describe the upper-body bilateral deficit (BD) in recreational climbers and to examine its relationship with specific performance. Fifteen recreational climbers (11 men, 4 women; 25–45 years; ≥3 years of experience) performed unilateral and bilateral maximal isometric handgrip strength test (HGT), and unilateral and bilateral maximal isometric strength test using a climbing-specific fingerboard test (CSFT). The fatigue resistance index test (FRI) and the endurance capacity test (ECT) were used as performance tests. Paired Student’s T-tests were used to compare bilateral deficit results between CSFT and HGT. Pearson’s correlation was used to assess relationships between these variables with the level at 5%. The results showed a significant (*p* < 0.05) bilateral deficit in the CSFT (−2.53 ± 4.49%) and the HGT (−2.05 ± 3.29%), with no difference between methods. A positive correlation was found between bilateral deficit in the CSFT and the ECT (r = 0.53; *p* = 0.044), while no correlation was observed with the FRI. Relative bilateral strength to body mass was strongly associated with ECT (r = 0.92; *p* < 0.001). Greater bilateral deficit was associated with endurance capacity in climbers. In addition, the CSFT demonstrated good predictive accuracy and may be considered a specific and reliable tool for assessing BD in climbers.

## 1. Introduction

Sport climbing has gained worldwide recognition, particularly after its inclusion in the Tokyo 2020 Olympic Games. This increased visibility has stimulated greater interest in the physiological, physical, and biomechanical determinants of sports climbing performance [1]. Climbing performance is a multifactorial construct determined by the interaction of technical, neuromuscular, and physiological factors, including maximal strength relative to body mass, local muscular endurance of the upper limbs and forearm muscles, and anaerobic capacity [1,2,3,4,5]. Among these factors, finger flexor strength has been consistently identified as a key predictor of performance, alongside forearm endurance and shoulder girdle stability. In addition, the capacity to sustain repeated intermittent isometric contractions under conditions of restricted blood flow is considered a limiting factor in longer and more physically demanding routes. Accordingly, climbers exhibit superior finger flexor endurance during intermittent isometric tasks compared with non-climbers, reflecting sport-specific neuromuscular and metabolic adaptations [6,7,8,9].

An important neuromuscular aspect for performance is the connection between neural and muscular aspects, often examined through the bilateral deficit (BD) [10]. The bilateral deficit refers to the reduction in strength produced during bilateral contractions compared with the summed strength produced during unilateral contractions performed with the same limbs [11]. The bilateral deficit is primarily explained by neural mechanisms, such as interhemispheric inhibition and reduced motor unit recruitment during bilateral contractions, resulting in lower strength compared to unilateral efforts [10,12]. Recent studies suggest that bilateral deficit may be associated with sport-specific performance and fatigue in predominantly unilateral sports such as judo, volleyball, and tennis [13,14,15]. For example, a bilateral deficit of −4.5 ± 7.1% was observed in judo athletes [16], whereas deficits ranging from −20% to −31% were reported in countermovement jumps in volleyball athletes [13], and values of −11.8 ± 6.7% were identified in tennis players, positively associated with speed and agility performance [15]. Additionally, BD may impair tasks requiring maximal strength or power, thereby compromising performance [16].

Thus, previous studies have shown that bilateral deficit is an important factor in sports performance and should be investigated across different sports. However, no study has specifically analyzed the presence or magnitude of bilateral deficit in climbers or its relationship with climbing-specific performance. Moreover, climbing involves simultaneous and coordinated actions of both upper limbs, which may influence the manifestation of bilateral deficit differently compared with predominantly unilateral sports [17]. However, climbing movements are rarely purely bilateral or symmetrical. During climbing, each hand frequently interacts with holds of different shapes, depths, orientations, and mechanical demands (e.g., crimp grips, slopers, pinches, pockets, or underclings), often requiring distinct strength magnitudes and grip configurations between limbs. Consequently, climbers commonly perform offset bilateral actions rather than identical bilateral contractions. These task-specific differences between limbs may alter neural coordination patterns, interhemispheric inhibition, and motor-unit recruitment strategies during strength production, potentially modifying the manifestation of bilateral deficit in climbing tasks. Therefore, it is necessary to investigate the presence of bilateral deficit and its relationship with performance in climbers. By the same token, handgrip dynamometry has been used to assess upper-limb strength, both unilaterally and bilaterally, in different studies [16,18]. It allows maximal isometric contractions with both hands simultaneously, making it possible to calculate the bilateral strength deficit and perform a specific bilateral assessment of grip strength [18]. Nevertheless, the ecological validity of the handgrip test in climbing has been questioned. For example, handgrip tests may not adequately reflect the specific demands of climbing [19], suggesting that fingerboard-based assessments combined with a load cell or electronic scale could provide greater precision in evaluating grip strength.

Therefore, the present study primarily aimed to examine the magnitude of the bilateral deficit in upper-limb strength and its association with climbing-specific performance in recreational climbers. A secondary aim was to determine the predictive accuracy of a climbing-specific fingerboard assessment for estimating upper-body bilateral deficit, using handgrip dynamometry as the reference method. We hypothesized that climbers would not exhibit a measurable bilateral deficit in the upper limbs and, consequently, that bilateral deficit would not be associated with performance in climbing-specific strength and endurance tests.

## 2. Materials and Methods

### 2.1. Participants

The sample consisted of 15 recreational climbers (11 men and 4 women; 32.67 ± 5.85 years; 70.02 ± 14.38 kg; 1.73 ± 0.105 m of height; 1.77 ± 0.13 m of wingspan, 23.23 ± 3.67 kg/m^2^ of BMI and 9.7 ± 5.0 years of experience). A convenience sample was recruited based on participant availability and eligibility during the data collection period. Additionally, no a priori sample size calculation was performed, which should be considered a limitation of the study. All participants suspended their training routines and were instructed to maintain their usual diet and hydration throughout the study. Volunteers signed informed consent forms, and the research protocol was approved by the University Research Ethics Committee (Protocol: 25632719.1.0000.5147). Volunteers presenting conditions that prevent intense physical activity, such as severe orthopedic disorders (particularly of the upper limbs), shortness of breath or chest discomfort during daily activities, or difficulty, pain, or dizziness when climbing stairs, were not eligible to participate in the study protocol. Moreover, individuals with recent major cardiovascular events (e.g., heart failure, uncontrolled hypertension), acute infections, or other illnesses were likewise not eligible, as were those taking medications that could affect muscle function or who answered “yes” to any item on the Physical Activity Readiness Questionnaire (PAR-Q). Participants were classified as recreational climbers because they did not participate in official climbing competitions or professional climbing activities. However, participants had previous climbing experience and reported maximum self-reported bouldering grades ranging from V4 to V12.

### 2.2. Experimental Design

Each participant visited the laboratory twice, with a minimum of 48 h between visits. During the first visit, participants were familiarized with the procedures. Anthropometric data (height, arm span, body mass, and forearm circumference) were also recorded at that time. In all experimental sessions, the same warm-up protocol was performed, consisting of arm and neck rotations, push-ups, a 10 s hang on a pull-up bar, a 10 s hang on a 20 mm wooden fingerboard (Woodtropia, Ouro Preto, Brazil, model Patrick and Poli 2) with feet supported, and finger slides. The familiarization procedures included handgrip strength testing and a subsequent climbing-specific fingerboard test (CSFT) setup. Randomization determined whether participants began with bilateral or unilateral CSFT, with three trials performed per condition and 1 min rest intervals between attempts. During unilateral testing, sides were alternated until three trials per limb were completed. Handgrip dynamometry followed the same randomized principle and was conducted separately. The load cell was calibrated between test conditions.

### 2.3. Anthropometric and Handgrip Assessment

Sample characterization included the assessment of body mass, height, arm span, and forearm circumference. Body mass was measured using a digital scale (Líder, Araçatuba, Brazil, model P-150M) with a resolution of 50 g, and height was determined with a stadiometer (Sanny, São Bernardo do Campo, Brazil, model Professional) with a resolution of 0.1 cm. Moreover, arm span and forearm circumference were measured according to the procedures described by Norton [20], and handgrip strength was assessed using a hydraulic dynamometer (Saehan, Masan, Republic of Korea, model SH5001). The procedure followed Turnes et al. [16] and adapted to the same positioning used for bilateral and unilateral climbing-specific fingerboard test (CSFT), i.e., shoulder flexed at 180°, full elbow extended, and forearm pronated.

### 2.4. Maximum Isometric Unilateral and Bilateral Climbing-Specific Fingerboard Test (CSFT)

**Figure 1:**
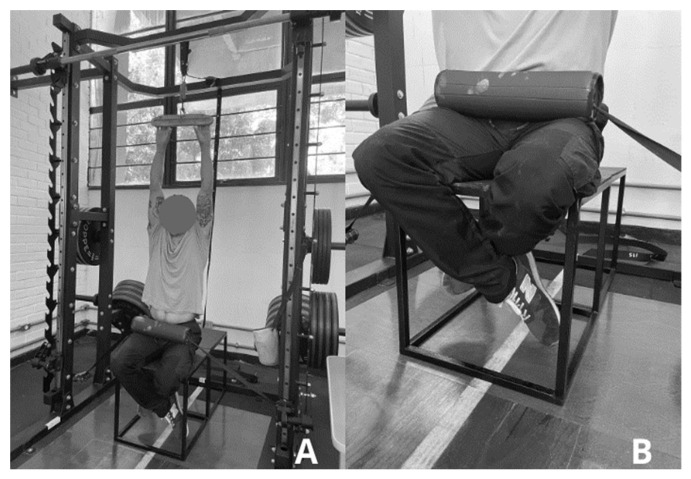
Arm (**A**) and legs (**B**) during CSFT.

Figure 1 illustrates the climbing-specific CSFT protocol. The protocol was standardized to ensure reliability and followed the procedures described by Labott et al. [21]. Both unilateral and bilateral CSFT were performed using a 20 mm fingerboard attached to a Progressor 200 load cell (Tindeq, Leknes, Norway, 200 kg capacity), previously validated by the same authors [21]. The values obtained from the load cell were expressed in kilogram-force (kgf). The load cells were calibrated before testing and between conditions. The grip type (half crimp or open hand) was chosen by the participant’s preference [22], without thumb assistance. However, body position was standardized with shoulder flexion at 180°, full elbow extension, and forearms in pronation [23,24]. The participants were instructed to flex their knees, keeping their feet below the bench without contacting the floor. During testing, verbal encouragement was provided to elicit maximal effort, and magnesium carbonate was applied to minimize hand slipping. Participants were seated beneath the frame supporting the load cell, and an adjustable restraining strap secured to the metal structure was used to limit lower-limb movement. The strap was padded with foam to ensure comfort. Three maximal 5 s trials were performed, separated by 1 min rest intervals, and the highest strength value was retained for analysis [25].

### 2.5. Fatigue Resistant Index Test (FRI) Endurance Capacity Test (ECT)

FRI was assessed during a 30 s maximal bilateral upper-body isometric strength test, following the same procedures as the CSFT. Specifically, a 20 mm fingerboard connected to a Progressor 200 load cell (Tindeq, 200 kg capacity), previously validated by Labott et al. [21], was used. The load cell was calibrated before testing. The grip type (half crimp or open hand) was chosen by the participant’s preference [22] without thumb assistance, and body position was standardized with shoulder flexion at 180°, full elbow extension, and forearms in pronation [23,24]. The participants were instructed to flex their knees, keeping their feet below the bench without contacting the floor, and to be seated beneath the frame supporting the load cell. An adjustable restraining strap secured to the metal structure was used to limit lower-limb movement. The highest strength values recorded during the first 5 s (Vmax) and the last 5 s (Vfinal) were used to calculate the fatigue resistance index, expressed as (Vfinal/Vmax) × 100.

### 2.6. Endurance Capacity Test (ECT)

The participants performed a bilateral dead hang at body weight on a 20 mm edge fingerboard hold for maximal duration. The grip type (half crimp or open hand) was chosen as the participant’s preference [22], without thumb assistance. Elbows were fully extended, and the shoulders were maintained in a controlled position to minimize swinging. The lower limbs were kept extended without contact with the ground or surrounding structures. The test was terminated when the participant voluntarily released the hold or when the feet touched the ground, as previously described by López-Rivera and González-Badillo [26]. The total time spent on the hold was recorded with a digital stopwatch and considered the endurance capacity (ECT), expressed in seconds.

### 2.7. Bilateral Deficit of Strength

The bilateral deficit of strength was calculated as the ratio between bilateral and unilateral performance in the protocols. This calculation utilized the right/left unilateral and bilateral values, as illustrated in Equation (1):

(1) $$bilateraldeficitofstrength\left(\%\right)\;=\;\left[100*\left(\frac{bilateral}{leftunilateral+rightunilateral}\right)\right]$$

Bilateral facilitation was identified when BD was significantly above 0, whereas bilateral deficit was noted when BD was significantly below 0. Bilateral deficit or bilateral facilitation was defined as a meaningful difference between performance in bilateral tasks and the combined results of unilateral tasks [12].

### 2.8. Statistical Analysis

Data are reported as mean ± SD. Normality was verified with the Shapiro–Wilk test, including the distribution of paired differences prior to paired comparisons. Paired Student’s t-tests were used to compare bilateral deficit results between CSFT and handgrip tests, and Pearson’s correlation to assess relationships between these variables. For the correlation analysis, we used the criteria proposed by Hopkins [27] to quantify the magnitude of the correlation, as follows: r = 0.0−0.10 (trivial), 0.10−0.29 (small), 0.30−0.49 (moderate), 0.50−0.69 (large), 0.7−0.89 (very large), and 0.9−1.0 (almost perfect). Moreover, test–retest reliability for bilateral and unilateral CSFTs across two sessions was analyzed using intraclass correlation coefficients (ICC 3,1) and typical error (TE). The interpretation of intraclass correlation coefficient (ICC) values was classified according to Koo and Li [28] as follows: >0.9 (excellent), 0.75–0.9 (good), 0.5–0.75 (moderate), and <0.5 (poor). All analyses were conducted in JASP (v.0.19.3), considering a significance level of 0.05 (i.e., *p* < 0.05).

## 3. Results

This study reported excellent reliability for all climbing-specific fingerboard tests (Table 1). For instance, the Bilateral CSFT fingerboard test showed ICC = 0.965 (95% CI: 0.900–0.988) with TE = 4.36 kg. Moreover, left unilateral CSFT yielded ICC = 0.969 (95% CI: 0.910–0.989) with TE = 2.16 kg, and right unilateral CSFT presented ICC = 0.931 (95% CI: 0.806–0.976) with TE = 3.26 kg.

Table 2 summarizes the descriptive results from all climbing-specific fingerboard tests and the handgrip test.

Table 3 summarizes the correlation values between bilateral deficit and performance tests. There were no differences between bilateral deficit measured from CSFT climbing-specific tests when compared to the CSFT measured from reference handgrip tests (t (14) = −0.351; *p* = 0.731). Additionally, there was a significant positive association between bilateral deficit estimated from CSFT and ECT (r = 0.526; *p* = 0.044 [large magnitude]), and relative bilateral CSFT and ECT (r = 0.919; *p* < 0.001 [almost perfect magnitude]). Nonetheless, no significant correlations were found between bilateral deficit estimated from CSFT and FRI (r = 0.258; *p* = 0.354 [small magnitude]), ECT and FRI (r = 0.240; *p* = 0.389 [small magnitude]), and relative strength and FRI (r = 0.098; *p* = 0.728 [trivial magnitude]).

## 4. Discussion

The primary aim of this study was to investigate the presence of the bilateral deficit in recreational climbers and to examine its relationship with performance variables. The main findings revealed indicate bilateral deficit values of −2.53 ± 4.49% in maximal isometric voluntary upper body pull test (CSFT) and −2.05 ± 3.29% in handgrip strength, with no significant differences between the assessment methods. These results suggest that the climbing-specific CSFT is a valid tool for measuring bilateral deficit in this population, while also showing that bilateral deficit values were lower than those typically reported in other sports. For instance, Turnes et al. [16] reported a bilateral deficit of −4.5 ± 7.1% in handgrip strength among judokas, and Pleša et al. [13] found deficits ranging from −20% to −31% in countermovement jumps among volleyball players, highlighting the variability of bilateral deficit depending on the neuromuscular demands of the sport.

Bilateral deficit is primarily of neural origin and has been attributed to mechanisms such as interhemispheric inhibition and reduced motor unit recruitment during bilateral contractions [10,12]. Although often associated with impaired performance in tasks requiring maximal strength, bilateral deficit is a plastic phenomenon that can be attenuated or even reversed into bilateral facilitation, depending on sport-specific demands. Evidence from other sports reinforces this variability. Kons et al. [14] reported a significant bilateral deficit in all countermovement jump parameters among elite judokas (r = −0.57 to −0.82) correlated with performance in the Special Judo Fitness Test. Similarly, Shu et al. [15] found average bilateral deficit values of −11.8 ± 6.7% in vertical and horizontal jumps among tennis players, which correlated positively with sprint and change-of-direction performance. Kozinc & Šarabon [29] also reported moderate correlations between higher bilateral deficit in jump tasks (−12.3 ± 9.4%) and change-of-direction performance in basketball and tennis athletes. Collectively, these findings suggest that in unilateral-dominant sports, bilateral deficit may serve as an adaptive mechanism, whereas in bilateral sports, its magnitude tends to be smaller [17].

Our initial hypothesis—that climbers would not exhibit bilateral deficit values and that no significant association would be observed between bilateral deficit and performance—was not supported. This indicates that climbing cannot be classified as a purely bilateral activity. The unpredictable nature of routes and boulders, both in training and competition, requires climbers to alternate between bilateral and unilateral demands, depending on the technical requirements of each movement. Consequently, training programs should incorporate both bilateral exercises, such as those performed with fingerboards or campus boards, and unilateral exercises to minimize potential imbalances. Analogously, Hartley et al. [30] indicate that climbers display marked bilateral asymmetries in finger flexor strength, with the dominant hand consistently generating greater strength even after controlling for dominance (mean difference = 4.1%). Their findings suggest that such imbalances are inherent to the specific demands of climbing rather than being explained by preferred climbing style or performance level. From a practical perspective, their results also highlight the importance of systematically monitoring unilateral strength to mitigate excessive asymmetries, which may have implications for both performance optimization and injury prevention [31].

No significant association was found between bilateral deficit and the fatigue resistance index (FRI), suggesting that localized finger flexor fatigue may not be directly related to bilateral deficit in this population. It is important to note that the FRI protocol lasted only 30 s, producing an average strength reduction of ~21%. Previous studies with similar protocols reported greater declines; for example, Michailov et al. [32] observed average fatigue indices of 24.67% (test) and 28.63% (retest), yet these values were not significantly associated with climbing ability. Longer or more demanding protocols may be more sensitive for exploring potential associations between bilateral deficit and fatigue in future research. Another relevant finding was the strong association between bilateral strength relative to body mass and endurance capacity (ECT), reinforcing relative strength as a key predictor of climbing performance, in line with previous studies [5,26]. Additionally, CSFT testing demonstrated excellent reliability (ICC > 0.93; TE ≤ 4.36 kg), corroborating previous work on the validity of fingerboard-based protocols using load cells [19,21]. This methodological robustness strengthens the reliability of the present findings.

Traditionally interpreted as a limitation, a bilateral deficit may also represent a functional adaptation in climbing. A small-magnitude deficit could reflect greater neural efficiency, energy economy, and enhanced motor control in technical movements, where differentiated strength application between limbs is often more relevant than maximal simultaneous output. In this sense, a bilateral deficit may help sustain endurance by reducing excessive overload and facilitating alternating arm efforts, a hallmark of climbing performance. This study is not without limitations. The relatively small sample size (*n* = 15) and the unequal sex distribution, with only four female participants, may limit the generalizability of the findings. In addition, although participants were classified as recreational climbers because they did not participate in official competitions, they presented heterogeneous climbing abilities, which may have influenced the variability of the results. Additionally, the relatively broad age range of the participants may have contributed to variability in neuromuscular performance outcomes. Furthermore, the laboratory-based assessments may not fully replicate the complex and dynamic demands of outdoor or competitive climbing. Additionally, no a priori sample size calculation was performed, which may have reduced the statistical power to detect smaller associations between variables. Future studies should include larger and more diverse samples, investigate climbers from different performance levels, and examine whether training interventions targeting bilateral deficits translate into improvements in climbing-specific performance and fatigue resistance. Additionally, future investigations should incorporate more ecologically valid climbing tasks and real-world performance measures.

## 5. Conclusions

In summary, recreational climbers exhibited a bilateral deficit of low magnitude, which nevertheless showed an association with endurance performance. Relative strength to body mass emerged as a finding of a more consistent predictor of climbing capacity. Taken together, these findings suggest that bilateral deficit should not be interpreted solely as a limitation, but also as a potential functional adaptation to the specific demands of climbing. Collectively, these findings indicate that CSFT assessment may serve as a complementary tool for performance measuring and monitoring in recreational climbers. Future research should compare competitive and recreational climbers, investigate the effects of targeted training interventions on bilateral deficit and determine whether changes in bilateral deficit are associated with improvements in climbing-specific performance, fatigue tolerance, and competition outcomes.

## Figures and Tables

**Table 1:** Mean ± SD and reliability values of the CSFT.

CSFT	Mean ± SD	ICC (95% CI)	TE (95% CI)
Bilateral (kg)	108.31 ± 22.23	0.97 (0.90–0.99)	4.21 (2.82–5.15)
Unilateral (Left, kg)	54.49 ± 12.06	0.97 (0.91–0.99)	2.16 (1.21–2.85)
Unilateral (Right, kg)	56.56 ± 11.96	0.93 (0.81–0.98)	3.27 (1.77–4.33)

CSFT = Climbing-specific fingerboard test; ICC = Intraclass Correlation Coefficient; TE = typical error; SD = standard deviation; CI = Confidence Interval.

**Table 2:** Descriptive values of climbing-specific and handgrip tests.

Variables	Mean ± SD	Minimum	Maximum
FRI (%)	78.99 ± 10.74	57.98	100.95
ECT (s)	37.87 ± 14.99	3.96	61.80
Bilateral Deficit CSFT (%)	−2.53 ± 4.49	−7.53	7.33
Bilateral Deficit Handgrip (%)	−2.05 ± 3.29	−7.58	3.80
Bilateral CSFT (kg)	107.53 ± 21.91	68.40	139.20
Unilateral CSFT (Left, kg)	55.13 ± 12.27	35.00	73.90
Unilateral CSFT (Right, kg)	56.95 ± 11.90	33.00	73.90
Bilateral CSFT relative (%)	158.33 ± 32.15	102.99	205.11
Unilateral CSFT relative (Left, %)	79.84 ± 16.12	53.85	105.35
Unilateral CSFT relative (Right, %)	82.32 ± 14.93	57.54	104.46
Bilateral handgrip (kg)	93.60 ± 20.55	55.00	137.00
Unilateral handgrip (Left, %)	47.33 ± 11.08	30.00	72.00
Unilateral handgrip (Right, %)	48.27 ± 10.88	26.00	73.00

CSFT = Climbing-specific fingerboard test; FRI = Fatigue Resistance Index; ECT = Endurance Capacity Test; SD = standard deviation.

**Table 3:** Correlation between CSFT bilateral deficit and bilateral CSFT relative to body mass, in relation to climbing-specific performance tests.

Variables	ECT (s)	FRI (%)
CSFT Bilateral deficit (%)	0.526 *	0.258
Bilateral CSFT relative (%)	0.919 *	0.098

CSFT = Climbing-specific fingerboard test; ECT = Endurance Capacity Test; FRI = Fatigue Resistance Index. * *p* < 0.05.

## Data Availability

The data are available upon requesting for the corresponding author.

## Abbreviations

The following abbreviations are used in this manuscript:

BD	Bilateral Deficit
CSFT	Climbing-Specific Fingerboard Test
HGT	Handgrip Strength Test
FRI	Fatigue Resistance Index
ECT	Endurance Capacity Test
ICC	Intraclass Correlation Coefficient
TE	Typical Error
SD	Standard Deviation
CI	Confidence Interval
